# Variability of Botulinum Toxins: Challenges and Opportunities for the Future

**DOI:** 10.3390/toxins10090374

**Published:** 2018-09-13

**Authors:** Christine Rasetti-Escargueil, Emmanuel Lemichez, Michel R. Popoff

**Affiliations:** 1 Institut Pasteur, Département de Microbiologie, Unité des Toxines Bactériennes, 25 Rue du Docteur Roux, 75015 Paris, France; christine.rasetti-escargueil@pasteur.fr (C.R.-E.); emmanuel.lemichez@pasteur.fr (E.L.); 2 Université Paris Diderot, Sorbonne Paris Cité, 75205 Paris, France

**Keywords:** botulinum neurotoxins (BoNTs), subtypes, BoNT variants

## Abstract

Botulinum neurotoxins (BoNTs) are the most potent known toxins, and are therefore classified as extremely harmful biological weapons. However, BoNTs are therapeutic drugs that are widely used and have an increasing number of applications. BoNTs show a high diversity and are divided into multiple types and subtypes. Better understanding of the activity at the molecular and clinical levels of the natural BoNT variants as well as the development of BoNT-based chimeric molecules opens the door to novel medical applications such as silencing the sensory neurons at targeted areas and dermal restoration. This short review is focused on BoNTs’ variability and the opportunities or challenges posed for future clinical applications.

## 1. Introduction

Botulinum neurotoxins (BoNTs) are among the most toxic known substances, whether of biological or chemical origin; they are part of the “dirty dozen” agents that are listed as possible bioweapons. BoNTs are produced by strains of the genus *Clostridium* comprising Gram-positive, anaerobic spore-forming bacterium *Clostridium botulinum*, other *Clostridium* spp. (*Clostridium butyricum* and *Clostridium baratii*), and a few non-clostridial species that produce 10 different toxinotypes of BoNTs causing botulism [1]. Botulism mainly consists of three naturally occurring forms: Foodborne botulism by oral intoxication, botulism by intestinal colonization (infant botulism and adult intestinal botulism), and wound botulism. Rare but often severe naturally occurring foodborne intoxications are still encountered worldwide as well as infant botulism cases due to intestinal colonization by *Clostridium* spores. Iatrogenic botulism cases due to the increased cosmetic usage of counterfeit and poor quality BoNT have also been reported. More rarely, inhalational botulism could result from the aerosolization of botulinum toxin. All of these cause the same clinical syndrome of symmetrical cranial nerve palsies followed by the descending, symmetric flaccid paralysis of voluntary muscles as well as the inhibition of secretions, which may progress to respiratory compromise and death. Treatment includes meticulous intensive care with mechanical ventilation when necessary and the administration of antitoxin. The antitoxins are mainly effective if administered early after the onset of symptoms [2,3].

BoNT/A is the deadliest biological substance currently known, with lethal dose values of 1 ng/kg in humans by the intravenous and subcutaneous routes [4]. Due to this extreme toxicity and ease of production, BoNT has been classified as a category A biothreat agent (by the United States Centers of Disease Control and Prevention) [5]. The Soviet Union and Iraq have weaponized BoNTs and attempted splicing the botulinum gene into other bacteria as reported by United Nations (U.N.) officers [6]. Furthermore, the risk of contamination of the food chain by BoNTs has been highlighted in several potential scenarios [7]. However, no effective attack by deliberate contamination by BoNTs has been reported to date.

Human botulism is mostly caused by BoNT/A, B, and E, while BoNT/F or C are involved in less than 1% of human botulism cases [8,9,10]. The BoNT/H was presented in 2014 as a new toxinotype of botulinum toxin, but further analyses suggested that this is a BoNT/A1 and /F5 hybrid-like BoNT which is neutralized by serotype A antitoxin [11,12,13]. In addition, BoNT/like toxins have been identified in non-clostridial species such as *Enterococcus faecium* and *Chryseobacterium piperi* [14]. The more recently discovered BoNT/x adds to the BoNT diversity as a recognized new novel toxinotype from *Weissella oryzae* that cleaves VAMP2 similarly to BoNT/B, D, F, and G [15,16]. Moreover, the activity of novel BoNT types remains to be defined. To add to this complexity, sequence analysis has allowed distinguishing the numerous variants within each BoNT toxinotype (more than 40) named “subtypes” (BoNT/A1-A8, BoNT/B1-B8 etc.) [1].

**Figure 1:**
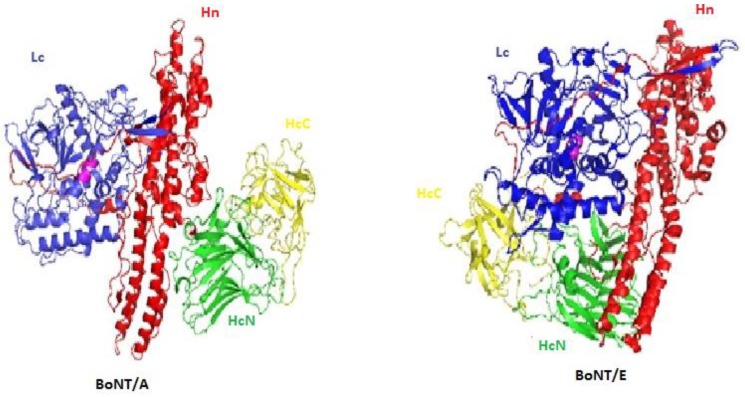
Structure of botulinum neurotoxins (BoNT)/A (pdb 3BTA) and BoNT/E (pdb 3FFZ): The catalytic domain (Lc) is colored in blue, the translocation domain (Hn) is colored in red, the N-terminal binding sub-domain (HcN) is colored in green, and the C-terminal binding sub-domain (HcC) is colored in yellow. The catalytic zinc site is depicted as a ball in magenta. Figures were produced with the program MacPymol.

BoNTs are produced as large protein complexes combining a neurotoxic subunit with a non-toxic non-hemagglutinin (NTNH) component, and for some toxin complexes, with either hemagglutinin (HA) components or OrfX proteins. Despite their apparent sequence complexity, BoNTs share a similar structure consisting of a light chain (Lc, 50 kDa) and a heavy chain (Hc, 100 kDa). The crystal structures of BoNT/A, /B, and /E, show a trimodular architecture with each domain fulfilling a ‘chaperone-like’ role for the other domains (Figure 1) [17,18]. The Lc is a zinc metalloprotease that cleaves one of the three SNARE proteins that are involved in neurotransmitter exocytosis; the N-terminal (Hn) domain assists the translocation of the L chain across the membrane of intraneuronal vesicles into the cytosol, while the half C-terminal (Hc) domain, which is composed of two sub-domains (HcN and HcC), is responsible for the binding of the toxin to the presynaptic membrane of neurons for endocytosis [19,20]. However, the precise mechanism by which Lc chains translocate through vesicular membranes into the cytosol remains to be defined.

BoNT is a sophisticated bioweapon with both toxic effects and therapeutic benefits, excluding massive vaccination as a widely applied preventative measure. While BoNTs have the potential to cause global harm, elucidating the features of their mode of action has led to increasing therapeutic applications of the neurotoxins to treat a wide range of conditions. Nevertheless, the understanding of the molecular underpinnings among all of the variants still remains to be elucidated [21,22].

This short review will focus on the BoNTs’ variability and the opportunities or challenges posed for future clinical applications.

## 2. Entry of BoNTs into Neuronal Cells and Other Cells

In most naturally acquired botulism forms, BoNTs enter by the oral route (foodborne botulism) or are produced directly within the intestine (infant or intestinal botulism) subsequent to *Clostridium botulinum* colonization, and undergo their transcytosis across the digestive mucosa [23,24]. After diffusion into the extracellular fluid and blood stream circulation, BoNTs target motor neurons’ endings. The entry of botulinum neurotoxins into neuronal cells is mediated through binding to a double-cell membrane receptor comprising a membrane glycoprotein and a ganglioside GD1b, GT1b, and/or GD1a [19,25,26,27]. The crystal structures of the Hc of BoNT/A1 [28], BoNT/B [29], and BoNT/F [30] bound to complex gangliosides GT1b or GD1a analogs provided further evidence for the existence of a conserved core ganglioside-binding site (SxWY) within BoNT/A, BoNT/B, BoNT/E, BoNT/F, BoNT/G, and tetanus toxin (TeNT) [31,32,33,34,35].

With regard to the protein part of the BoNT receptor complex, BoNT/A, C, E, and F bind to the luminal domains of either of the three isoforms of the vesicle protein SV2, while BoNT/B, DC, and G bind to synaptotagmin I or II (Syt) [33,36,37,38,39]. The results obtained for BoNT/B and BoNT/G with deactivated protein receptor binding sites in phrenic nerve preparations containing or deficient in complex gangliosides confirmed this conclusion [27].

A high-resolution crystal structure of full-length BoNT/B revealed that the helix of Syt II binds to the C-terminal end of HcC; this locus is adjacent to the non-overlapping ganglioside-binding site of BoNT/B. Binding to the ganglioside GT1b also enables BoNT/B to sense a low pH for directing the formation of a translocation channel [40].

Thus, the high affinity of BoNTs for presynaptic membranes presumably results from multiple synergistic interactions with the ganglioside and protein parts of the receptor [26].

In addition, interactions with the membrane receptors differ according to BoNT types.

For example, the crystal structure of BoNT/C-Hc revealed a sialic acid binding site that was different from the established ganglioside-binding sites, suggesting one or more additional potential ganglioside or carbohydrate-binding sites for this toxinotype. Based on these findings, it has been speculated that BoNT/C might bind to neurons via multiple gangliosides and/or phospholipids [41].

Furthermore, the mosaic toxin BoNT/DC does not contain the consensus ganglioside binding site, but does interact with lipid membranes by extending a loop recognizing sialic acid-containing moieties, as shown in liposome flotation assays. Binding avidity to the membrane is further enhanced thanks to the protruding loop that anchors the toxin into the plasma membrane [42]. More recently, elegant approaches of toxin binding to nanodisc-embedded receptors combined with surface plasmon resonance suggest that the anchoring of the Hc loop into the lipid bilayer compensates the entropic penalty of a double receptor-binding configuration [43].

In addition to the Hc role as a whole, the coordination between the Hc and Hn domains is deemed to be very important for the efficient translocation of the L chain [18,20,44]. Yet, little is known about the Hn domain function, except that the Hn domain is thought to be important for orienting the Lc and forming channels across the endosomal membrane [45]. HcN is known to adopt a β-sheet jelly-roll fold, binding to the microdomains of the plasma membrane and interacting with phosphatidylinositol phosphates [17,46]. An acidic environment inside the vesicles provokes a transition into the structure of Hn that forms a channel sustaining the translocation of the unfolded Lc chain across vesicular membranes [47,48]. These results strongly indicate that the toxin binding to the neuronal cell wall does not reside solely in the Hc domain, but that the HcN domain actively participates in the binding, and this participation is vital for the neurotoxicity to develop. Nevertheless, the functional role of the Hn domain in the multiphasic action of BoNTs and the molecular mechanism of translocation remain to be clarified.

Whatever the considered clostridial neurotoxin, the identified protein receptors are expressed on several cell types, including intestinal crypt epithelial cells (binding of the toxin to cells or the defined existence of a receptor) [49]. However, BoNTs’ receptors identified at the intestinal epithelial cells mainly consist of gangliosides or related glycoprotein. The entry of BoNTs into intestinal cells is somehow following specific pathways, since BoNT/A and BoNT/B transcytosis through intestinal cells requires a specific pool of actin filaments controlled by Cdc42 [50,51]. Contrary to their intraneuronal traffic, the BoNTs follow non-acidic compartments within intestinal cells, enabling the release of whole undissociated and functional toxins at the basolateral side.

**Figure 2:**
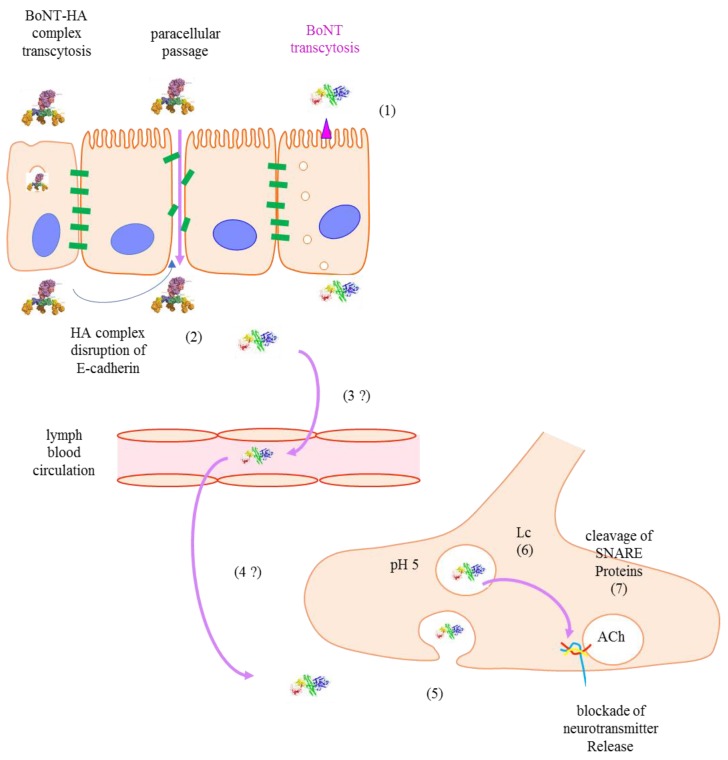
Route and mechanism of intoxication by BoNT. (1) BoNTs and BoNT-hemagglutinin (HA) are transcytosed through epithelial and microfold (M) cells respectively in human intestinal epithelia; (2) In the extracellular region, the trimeric HA complexes bind to E-cadherin, leading to intestinal barrier disruption and paracellular passage; (3 and 4) BoNTs enter into the circulatory system and reach nerve terminals by an unknown way; (5) BoNTs bind to polysialoganglioside (PSG) and synaptic vesicle glycoprotein 2 (SV2); and enter neurons via clathrin-dependent endocytosis; (6) Hn domains form translocation channels for Lc to escape the endosomal acidic environment toward the neutral cytosol where the Lc is activated as a Zn^2+^-dependent protease; (7) The Lc protease cleaves SNAP-25 with extremely high specificity to block the synaptic vesicle traffic and neurotransmitter release.

In addition, BoNT partly passes via the paracellular route, since the HA complex, at high doses, has been found to perturb the E-cadherin intercellular junctions between epithelial intestinal cells. It is noteworthy that the uptake of BoNTs alone or as complexes through the intestinal barrier is poorly efficient [51,52,53] (Figure 2).

However, based on recently published data, it is clear that BoNTs have a wider zone of influence than originally described on neuronal cells, including epidermal keratinocytes, mesenchymal stem cells from subcutaneous adipose tissue, nasal mucosal cells, urothelial cells, intestinal epithelial cells, neutrophils, macrophages, and prostate, breast, and alveolar epithelial cells. Several types of non-neuronal cells may be directly affected by BoNT, producing a biological effect arising from the cholinergic output disruption by BoNTs. BoNT/A can also elicit specific biological effects in dermal fibroblasts, sebocytes, and vascular endothelial cells. It has been recently shown that BoNT/A protects skin flaps, facilitates wound healing, decreases the thickness of hypertrophic scars, produces an anti-aging effect, and improves a mouse model of psoriasiform dermatitis. These new findings lay fertile ground for future studies that are likely to support useful discoveries in terms of therapeutic uses of BoNTs [54].

## 3. Susceptibility to Different Botulinum Neurotoxin Toxinotypes and Subtypes

While humans are mainly susceptible to BoNT/A, B, or E, susceptibility to the different BoNT toxinotypes varies among animal species. Small rodents such as mice are highly susceptible to all of the BoNT types. Albeit cattle are susceptible to type A and B, most outbreaks are caused by BoNT types C, D, or D/C. The mosaic *C. botulinum* C/D and D/C strains seem to be widely distributed in mammals, birds, and the environment [55,56]. *Clostridium argentinense* that produces BoNT/G has been found in soil, but no naturally acquired BoNT/G botulism has been recorded in human or animal. In addition, the geographical distribution of BoNT-producing clostridia is variable. The toxinotypes A and B occur more frequently in soil samples, and the regional distribution of these two toxinotypes is different. For example, toxinotype A is predominant in the western United States, whereas toxinotype B prevails in the eastern United States, as well as in Europe. Toxinotype E is more predominant in sea or lake sediments and fish than in the soil of northern countries [57].

In addition to this differential animal susceptibility, the onset, duration of paralysis, and severity of the symptoms significantly varies among each BoNT toxinotype. All of the BoNTs types act by targeting the same vesicle fusion machinery (consisting of SNARE complexes including VAMP/synaptobrevin, synaptosomal-associated protein (SNAP-25), syntaxin, and additional proteins), but cause paralysis of different durations as a result of different intracellular behaviors, and in particular due to a variable persistence of the cleaved Lc within the cytosol of intoxicated neurons [58]. BoNT toxinotypes B, D, F, and G specifically cleave the vesicle associated membrane protein (VAMP) synaptobrevin, at different peptide bonds. BoNT types A, C, and E cleave SNAP-25 at different sites located within the carboxyl terminus, while BoNT type C additionally cleaves syntaxin. In a mouse study by Keller et al., the running activity of mice injected with BoNT/A, B or E revealed that complete and partial paralysis persisted in the order of A > B > E, in agreement with the results from previous studies [59]. Considering its high sensitivity, the mouse model is a good predictor of toxin action in human tissue. The presence of a dileucine motif in the Lc of BoNT/A seems responsible for its long-lasting duration of action in motor nerves due to the persistent truncation of synaptosomal-associated protein (SNAP-25) [60]. However, the lifetime of Lc in neuronal cells is probably not the only factor involved in the duration of BoNT effects. Indeed, SNARE complexes likely adopt a radial arrangement, and this supports the idea that SNAP-25, once it is cleaved by BoNT/A, does not impair the SNARE complex assembly, but rather acts as a dominant negative SNARE, thereby causing a long duration of blocking effect on the neuroexocytosis machinery [61,62]. As a consequence, BoNT/E has not been used to treat human muscle disorders because of its shorter duration of action compared to BoNT/A and B, but may be beneficial in treatments requiring short-acting BoNTs [63].

For BoNT/A, there are currently eight known subtypes (BoNT/A1-A8) that share between 84–97% sequence identity, and may have different neurotoxic activities, although only functional differences between BoNT/A1 and /A2 have been identified to date [64]. The most widely used therapeutic BoNT is the subtype A1, due to rapid paralysis onset and long-lasting effects. Other BoNT/A subtypes exhibit different properties to BoNT/A1 such as the onset of symptoms and duration of action [65,66,67]. In neuronal cells models, HcA2 displayed a higher degree of receptor occupancy than HcA1, and a stronger inhibitory effect on neurotransmitter release [68] (and Rasetti–Escargueil unpublished data). Moreover, differences in the initial interaction of HcA1 and HcA2 with neurons may contribute to the development of unique pathologies due to BoNT/A1 and BoNT/A2: BoNT/A2 triggers a faster and increased intoxication of neuronal cells compared to A1 due to faster cell entry by BoNT/A2, while different paralytic symptoms are observed in mice [64]. The BoNT/A3 has also been shown to be less effectively neutralized by anti-BoNT/A1 antibodies, whilst also inducing symptoms of intoxication in mice that are distinct from BoNT/A1. It has been suggested that these differences are due to structural differences within the binding domain of BoNT/A3 (Hc/A3) [66]. Subtype differences of the Hc domains from BoNT/A3 and BoNT/A4 provide a plausible explanation of how the structural differences may play a role in receptor binding and the subsequent different symptoms observed. Indeed, the mutations evidenced on the BoNT/A3 and A4 binding domains explain their differential binding mode [69].

## 4. Implications for the Future Clinical Applications and Antibodies Development

Botulism, which was initially coined as sausage poisoning or “Kerner’s disease”, was first described in a manuscript published in 1822 where Kerner summarized 155 cases of “sausage poisoning” in Germany [70]. Remarkably, he also envisioned the possibility of using this paralyzing poison to treat diseases associated with overactive nervous system symptoms, including muscle hypercontraction and the hypersecretion of body fluids.

BoNT/A is used in the clinic for a growing number of indications, including skeletal and smooth muscle disorders, hypersecretory glands disorders, and nociceptive pain mechanisms [71,72]. Although initially thought to inhibit acetylcholine release only at the neuromuscular junction, BoNTs are now recognized as an efficient blocker of the peripheral release of the neurotransmitters involved in pain regulation. Initially used to treat strabismus in order to avoid surgery side effects, BoNT has now more than 100 possible medical applications, including movement disorders, hemifacial spasm, essential tremor, tics, writer’s cramp, cervical dystonia, cerebral palsy, and vascular cerebral stroke [73] (Table 1). More recently, BoNT has been approved for chronic pain, migraine headaches, overactive bladder, inflammation, and remarkably, some forms of depression, opening new avenues to search for new BoNT applications to treat behavioral disorders. Although initially thought to inhibit acetylcholine release only at the neuromuscular junction, BoNTs are now recognized to inhibit acetylcholine release at autonomic cholinergic nerve terminals, as well as blocking the peripheral release of neurotransmitters involved in pain regulation.

The primary reason for the increased therapeutic application of BoNT/A is due to its marked prolonged clinical efficacy and proven safety record [74]. However, the wide range of BoNT/A dosage used in medical or cosmetic applications or the use of poorly characterized products bears a substantial risk of accidental overdosage. The BoNT/A1–SV2C complex crystal structure data, combined with the results of the binding differences between subtypes, could provide a strong platform for the design of novel BoNT/A1 variants with attenuated binding properties, allowing safer applications of the toxin by reducing the risks of overdosage [75]. Moreover, the engineering of BoNTs with reduced functions is a potent strategy for the development of vaccines against botulism and other toxin-mediated diseases [76].

The flaccid paralysis caused by BoNT-induced peripheral cholinergic neuron paralysis versus the central inhibition of glycinergic neurons caused by TeNT is the classical picture indicating that BoNTs do not undergo retrograde transport along axons contrarily to TeNT. Nevertheless, the experimental work by Caleo et al. has evidenced the significant retrograde transport and transcytosis of BoNTs across neurons in vivo up to the central nervous system, despite the classical distinction between botulinum and TeNT toxins establishing that BoNTs remained localized to the injection site unlike TeNT [77,78,79]. As a consequence, a more detailed understanding of the central trafficking and actions of BoNT is now required in particular for its potential therapeutic applications to human epilepsy and pain management [80,81].

Another one of the clinical unexpected effects of BoNTs is the positron emission tomography (PET) study of the cortical activation patterns induced by BoNT injections to treat dystonia patients. Indeed, BoNT injections improved their writing technique together with increased activation in the parietal cortex and caudal supplementary motor area. These results demonstrated for the first time a cortical reorganization that was secondary to the BoNT-induced changes in motor strategy. Changes in peripheral feedback or its central integration play a major role in the pathophysiology of movement disorders [82]. Hence, the long-term clinical benefits of BoNT treatment could also reflect plastic changes in motor cortex output after the reorganization of the whole synaptic circuitry, and more generally support the need to explore intraneuronal transport and the central effects of BoNTs more closely [83,84]. The central effects of BoNT are further supported by their application in writer’s cramp or Meige’s syndrome, in which BoNT likely induces a reorganization of cortical somatosensory circuits instead of a unique activity on peripheral motoneurons [85,86,87].

Other potential applications of BoNT include the targeting to non-cholinergic nerves and non-neuronal cells for the treatment of hypersecretory disorders (e.g., cystic fibrosis) or hormonal disorders (e.g., acromegaly). BoNT is able to suppress the effects of parasympathetic nerves on exocrine glands and smooth muscle, while suppressing the effects of cholinergic sympathetic nerves to sweat glands. These actions impart the ability of BoNT to affect autonomic disorders such as axillary or plantar hyperhidrosis. Urogenital disorders, hyperactive bladder, and benign prostatic hypertrophy are similarly effectively treated with BoNT. BoNT is likewise useful in a variety of pain disorders mainly by inhibiting the neuronal release of nociceptive amino acids, peptides, and inflammatory mediators. Engineered BoNT has also been proposed for treating botulinum poisoning. Hence, there is a growing expectation for major advances in BoNT neuroprotective strategies and for the development of newly targeted (extraneuronal) BoNT-based chimeric toxins as therapeutics [88].

Another method may rely on the engineering of novel BoNT/A pharmaceuticals for the treatment of diseases that rely on SNAP-23-mediated hypersecretion. Of note, it has been found to be desirable to engineer LcA variants that are able to cleave hSNAP-23 as a potential therapeutic for chronic obstructive pulmonary disease and asthma. The systematic mutagenesis of two major BoNT/A binding pockets was already conducted in order to adapt these pockets to the corresponding amino acids of human SNAP-23. Human SNAP-23 cleaving mutants were isolated using an elegant yeast-based screening approach [89].

In addition to adapting the binding pockets, the differential receptor binding of subtypes might explain the differences in biological activity, and may support the design of new therapeutic neurotoxins [63]. The structures of BoNT/A3 and A4 proteins share a high degree of similarity with other known BoNT Hc domains, whilst containing some subtle differences that support the differential binding hypothesis that may be exploited to develop therapeutic neurotoxins with novel therapeutic properties [69].

Beyond BoNTs-based therapies, a more in-depth knowledge of the subtype’s differences will enable the development of more efficient antibody therapies, since the capacities of antibodies greatly differ between subtypes. In the European program “AntiBotABE”, six immune phage display libraries were constructed, starting from macaques (Macaca fascicularis) immunized with a non-toxic recombinant fragment corresponding to BoNT/A1-Hc, BoNT/A1-Lc, BoNT/B2-Hc, BoNT/B2-Lc, BoNT/E3-Hc, and BoNT/E3-Lc. Antibodies that cross-neutralized BoNT/A1 and A2 and BoNT/B1 and B2 subtypes were selected, but with added complexity to adapt each evaluation method to each subtype. Such cross-neutralizing antibodies are highly sought after in therapy to expand the therapeutic spectrum while decreasing the number of antibodies that are required to develop a wide spectrum neutralizing mixture [90].

## 5. Concluding Remarks and Perspectives

BoNTs are the most toxic substances found in nature that may be used as bioweapons, but minute doses of this deadly poison are increasingly used in therapy, excluding vaccination, as a widely applied preventative measure. Thereby, BoNTs became mostly attractive therapeutic tools with numerous and various medical applications to be further developed rather than a bioweapon used for bioterrorist attacks, of which only a few unsuccessful attempts have been reported.

While elucidating the features of BoNTs’ mode of action led to increasing applications of these neurotoxins in therapy, the understanding of biochemical mechanisms underpinning the differences between variants still remains to be fully elucidated.

As described above, the susceptibility to the different BoNT toxinotypes varies among animal species, but the onset and duration of paralysis also significantly varies among each BoNT toxinotype in human or animal studies. The seven BoNTs all act by targeting the vesicle fusion machinery, but cause the paralysis of different durations as a result of different intracellular behaviors.

The BoNT crystal structure data combined with the results on the binding differences between subtypes will provide a strong platform for the design of safer BoNT variants with attenuated properties representing promising candidates for novel specific applications of the toxin, such as the silencing of sensory neurons at specifically targeted territories.

Moreover, novel biophysical approaches that allow one to follow the action of a single molecule and a single cell will shed light on the molecular mechanism underlying the action of specific BoNTs subtypes and support the identification of more efficient countermeasures.

## Figures and Tables

**Table 1:** Timeline for botulinum neurotoxins (BoNT).

Timeline	Name/Affiliation	Event
**886 AD**	Emperor Leo IV	banned ‘blood sausage’ as it caused fatal illness
**1822**	Justinus Kerner	sausage poison (possible therapeutic use)
**1870**	Müller	botulism (Latin: botulus) for sausage
**1895**	Van Ermengem	*Clostridium botulinum* (causative agent of botulism)
**1919**	G.S. Burke	determination of minimum lethal dose in guinea pigs
**1928**	Herman Sommer	BoNT (purified) isolation
**1946**	Carl Lamanna Edward Schantz	LD50 test: neurotoxin activity BTA in in crystalline form
**1949**	Arnold Burgen	neuromuscular transmission blockade
**1950**	Vernon Brooks	BoNT/A: blockade of acetylcholine from motor nerve endings
**1960s**	Schantz/Scott	strabismus: monkeys
**1980**	Scott	strabismus: humans
**1984**	FDA	designates BoNT/A for blepharospasm in adults
**1986**	FDA	designates BoNT/A for cervical dystonia
**1987**	Drs. Jean and Alastair Carruthers	Cosmetic benefits of BoNT/A found accidentally by ophthalmologists treating patients for involuntary blinking
**1988**	Allergan	Oculinum (BoNT/A): clinical trials
**1989**	FDA Allergan	Oculinum (BoNT/A): strabismus, blepharospasm, hemifacial spasm and dystonia (~25–40 ng/100 mouse LD_50_) Allergan buys Oculinum and renames as Botox
**1993**		SNAP-25: molecular target of botulinum toxin type A
**1995**	MHRA	approves Dysport^®^ (Ipsen)(~5 ng/500 mouse LD50) for strabismus in UK
**2000**	FDA	approves Botox^®^ (Allergan) for cervical dystonia
**2001**	-CPMP	Botox^®^ approval for cosmetic procedures in Canada and New Zealand. approves first type B toxin, NeuroBloc^®^ for cervical dystonia
**2002**	FDA	approves Botox^®^ for cosmetic therapy (Australia, Switzerland, Taiwan, and Singapore)
**2003**	AFSSAPS	approves Botox as Vistabel^®^ (France)
**2003**	FDA	approves Myobloc^®^ (Solstice Neuroscience) for cervical dystonia Neurobloc^®^
**2004**	FDA	approves Botox for primary axillary hyperhidrosis (severe underarm sweating)
**2006**	MHRA	approves Botox as Vistabel^®^ for treatment of glabellar lines
	-	Xeomin^®^ (Merz) licensed in Germany for blepharospasm and cervical dystonia in adults
**2006**	Korean FDA	Neuronox^®^ (Medy-Tox) approval for blepharospasm
**2009**	MHRA	approves Azzalure^®^ for treatment of glabellar lines
**2010**	FDA	approves Botox^®^ to treat chronic migraine
**2011**	FDA	approves Botox to treat specific form of urinary incontinence: bladder detrusor over-activity in patients with neurologic conditions
	FDA	approves Xeomin (incobotulinumtoxinA) as Bocouture^®^ for cosmetic uses in adult patients
**2012**	NHS UK	approves Botox^®^ to treat chronic migraine
**2013**	Thailand	approves Nabota^®^ (Daewoongs Pharmaceuticals)
**2014**	China	BTX-A product also approved as Lantox^®^ and Prosigne^®^ (Lanzhou Institute of Biological Products, China)
**2015**	FDA	FDA Approval of Xeomin (incobotulinumtoxinA) for adult upper limb spasticity
**2017**	FDA and EMEA	approve Botox^®^ and Dysport^®^ to treat adult upper and lower limb spasticity and Dysport^®^ only to treat children lower limb spasticity
**2018**	FDA	FDA approves Xeomin^®^ for sialorrhea

FDA: Food and Drug administration; MHRA: Medicines and Healthcare products Regulatory Agency; CPMP: Committee for Proprietary Medicinal Products; AFSSAPS: French Agency for the Safety of Health products (now ANSM, Agence Nationale de Sécurité du Médicament); EMEA: European Medicines Agency (Modified from Hanchanale et al. [73]).
